# Challenging assumptions: “unveiling meritocracy’s reality in neurosurgery”

**DOI:** 10.3389/fsurg.2024.1423999

**Published:** 2024-07-16

**Authors:** Manuel De Jesus Encarnacion Ramirez, Ismael Antonio Peralta Baez, Gervith Reyes Soto, Jeff Ntalaja Mukengeshay, Cherubin mpoyi tshiunza, Andreina Rosario Rosario, Nikolenko Vladimir Nikolaevich, Renat Nurmukhametov, Siddarth Kannan, Keith Simfukwe, Luis Miguel Duchén Rodríguez, Gennady Chmutin, Egor Chmutin, Albert Sufianov, Jose Antonio Soriano Sanchez, Andreas K. Demetriades, Matias Baldoncini, Alvaro Campero, Gennadii Piavchenko, Juan Carlos Roa Montes de Oca, Kazadi Kelvin Kalangu, Alistair Jenkins, Jesus Lafuente

**Affiliations:** ^1^ Department of Neurosurgery, Russian People’s Friendship University, Moscow, Russia; ^2^ Department of Neurosurgery, Hospital Regional Alejandro Cabral, San Juan de la Maguana, Dominican Republic; ^3^ Department of Head and Neck, Unidad de Neurociencias, Instituto Nacional de Cancerología, Mexico City, Mexico; ^4^ Department Neurosurgery, Clinique Ngaliema, Kinshasa, Democratic Republic of Congo; ^5^ School of Medicine, Autonomous University of Santo Domingo (UASD), Santo Domingo, Dominican Republic; ^6^ Human Anatomy and Histology Institute of Clinical Medicine N.V. Sklifosovsky FSAEI HE I.M., Sechenov First Moscow State Medical University (Sechenov University), Moscow, Russia; ^7^ School of Medicine, University of Central Lancashire, Preston, United Kingdom; ^8^ Department of Neurosurgery, Maina Soko Medical Center, Lusaka, Zambia; ^9^ Center for Neurological Diseases, Public University of El Alto, La Paz, Bolivia; ^10^ Department of Neurosurgery, I.M., Sechenov First Moscow State Medical University (Sechenov University), Moscow, Russia; ^11^ Departments of Neurosurgery, “Federal Centre of Neurosurgery” of Ministry of Health of the Russian Federation, Tyumen, Russia; ^12^ Spine Clinic, The American-British Cowdray Medical Center I.A.P, Mexico City, Mexico; ^13^ Department of Neurosurgery, Royal Infirmary, Edinburgh, United Kingdom; ^14^ Laboratory of Microsurgical Neuroanatomy, School of Medicine, University of Buenos Aires, Buenos Aries, Argentina; ^15^ Department of Neurosurgery, Hospital Padilla de Tucuman, San Miguel de Tucuman, Argentina; ^16^ Department of Human Anatomy and Histology, Sechenov University, Moscow, Russia; ^17^ Deparment of Neurosurgery, Complejo Asistencial Universitario de Salamanca, University of Salamanca, Salamanca, Spain; ^18^ Department of Neurosurgery, College of Health Sciences, University of Zimbabwe, Harare, Zimbabwe; ^19^ Department of Neurosurgery, Royal Victoria Infirmary, Newcastle upon Tyne, United Kingdom; ^20^ Department of Neurosurgery, Hospital Universitario del Mar, Barcelona, Spain

**Keywords:** meritocracy, neurosurgery, training, access, equality

## Abstract

**Introduction:**

Meritocracy, a concept revered as the cornerstone of fairness and equal opportunity, is critically examined in the context of neurosurgery. This article challenges the notion that success in this demanding field is solely determined by individual abilities and effort. It reveals that factors such as background, gender, and socioeconomic status significantly influence one's career trajectory. By investigating how these systemic barriers impact admissions to neurosurgical training programs and professional advancement, the paper underscores the complexity of meritocracy in neurosurgery, suggesting that the meritocratic ideal is more nuanced and influenced by external variables than commonly believed.

**Results:**

Certain universities deemed elite offer a curriculum divergent from that of their counterparts in low and middle-income countries. Students at these “elite” institutions gain exposure to new technologies and research incentives, which brings us to the realm of research. Remarkably, 75% of articles originating from developed nations account for just 25% of traumatic brain injury cases. This disparity highlights a significant research imbalance, and the common refrain underscores the need to bolster research capabilities in low-income countries. For neurosurgeons in the developing world, engaging in research often becomes a luxury due to multifaceted challenges. Financial barriers, including publication costs and paywalls for accessing articles, pose significant hurdles. Comparing salaries between countries underscores the glaring divide according to “Neurosurgeon Salary” in 2024. Neurosurgeons in the United States receive a median salary of $412,000 dollars per year, compared to $13,200 dollars in Latin America, as of June 2023. Given such incongruities, the prospect of even attending conferences or workshops abroad remains difficult for neurosurgeons from developing nations. Research isn't cast aside due to a lack of interest but due to resource limitations. The present landscape demands reconsideration.

**Conclusion:**

We underscore the journey towards a more inclusive and equitable future in neurosurgery as not just a goal, but a dynamic process fuelled by resilience, collaboration, and a commitment to diversity. The narrative promotes a collective endeavour to dismantle barriers and embrace innovation, emphasizing the importance of mentorship, cross-institutional collaboration, and the amplification of underrepresented voices.

## Introduction

Meritocracy, a concept revered as the cornerstone of fairness and equal opportunity, has long been an aspirational ideal in various fields, including the realm of neurosurgery. The belief that success is determined solely by one's abilities and effort, rather than factors like background, gender, or socioeconomic status, has fueled the idea that those who rise to prominence in this demanding field do so based solely on their merit (1). However, a closer examination of the intricacies within the realm of neurosurgery uncovers a more complex narrative, one that challenges the assumed meritocratic nature of the discipline. Neurosurgery, with its demanding nature and high stakes, has historically been viewed as a meritocratic field, where only the best and brightest succeed. Admissions to neurosurgical training programs often hinge on academic achievements, research accomplishments, and clinical skills. This rigorous selection process seemingly underlines the principle of meritocracy. However, it's essential to recognize that merit itself is often influenced by factors that are far from equal.

Is the concept of meritocracy truly a fallacy? It's a complex question, one that becomes increasingly challenging, if not impossible, when attempting to compare neurosurgeons and trainees who haven't had the same opportunities presented to them. While the quote “I worked hard” echoes frequently, it is pertinent to acknowledge that personal effort, though substantial, might not solely account for success (2, 3). Often, support and access play pivotal roles in determining outcomes. It's this support that needs to be extended to those who find themselves devoid of such opportunities. Undeniably, exceptional neurosurgeons have emerged, creating inspirational success stories that seem to underline the concept of meritocracy. However, these stories arguably remain the exception rather than the rule. Many groups continue to be underrepresented due to systemic barriers rooted on various social identifiers, including gender, race, ability, geographical location, and more. In exploring the diversity of neurosurgery, we encounter numerous examples of individuals from different ethnic origins and nationalities who have greatly enriched the field. Notably, several accomplished neurosurgeons were immigrants or geographic transplants, uprooting themselves from their native countries.

Gazi Yasargil, the groundbreaking neurosurgeon who revolutionized microneurosurgical techniques, was born in Turkey (4). Renowned Barrow Neurological Institute surgeons Robert Spetzler and Volker Sonntag immigrated to the United States from Germany during their childhood (5). Alfredo Quinones-Hinojosa, who initially crossed the Mexico–United States border to work on a farm, went on to become a Professor of Neurosurgery and Oncology at Johns Hopkins University, eventually chairing Neurosurgery at the Mayo Clinic in Jacksonville, Florida (6). Other notable geographic transplants in neurosurgery include Jacques Morcos (Lebanon), Raymond Sawaya (Syria, Lebanon) (7) Ossama Al-Mefty (Syria) (8), Kazadi Kalangu, a Congolese neurosurgeon who moved to Belgium and helped the development of modern neurosurgery in Zimbabwe; and many more. Perhaps their immense triumphs are a testament to the resilience, strength, and adaptability forged through their immigrant experiences.

In evaluating the notion of meritocracy in neurosurgery, it's crucial to acknowledge that personal determination is just one facet of success. Support, opportunities, and systemic factors must be taken into account, particularly given the barriers many underrepresented groups face. The awe-inspiring narratives of immigrant neurosurgeons underscore the multidimensional nature of achievement, unveiling the inherent disparities beneath the surface of apparent meritocracy. Moreover, the very definition of merit itself is worthy of scrutiny. Can merit truly be separated from context and circumstance? The pursuit of meritocracy assumes a level playing field, which often isn't the case.

The opportunities available to an individual are influenced by their upbringing, education, access to resources, and the network they're born into. A person's background can determine the avenues available to them, affecting their ability to display their talents and merit. It's also important to note that the idea of merit can be subjective, shaped by societal norms and biases. What one person or institution considers as merit might not align with another's perspective. Factors such as cultural bias, unconscious prejudice, and stereotyping can each unconsciously influence how merit is perceived and rewarded (7, 8). While the meritocratic ideal may be inspiring, its realization is complex, complicated and often elusive, especially in fields like neurosurgery. The exceptional achievements of a few individuals should not be taken as indicative of a level playing field. The experiences of immigrant neurosurgeons, who have transcended barriers through their remarkable journeys, emphasize that success often relies on a blend of talent, resilience, and a supportive environment. To genuinely foster meritocracy, systemic barriers must be recognized, then dismantled, and opportunities must be made accessible to all.

### A global imbalance: addressing disparities in neurosurgical advancement is the challenge of inequity in neurosurgical advancement a regional problem?

The term “third world” (low-income countries) inadequately captures the complexity; we're better off referring to these regions as developing countries or those with low income. Within these contexts, an issue emerges known as “academic aristocratization,” akin to academic elitism. Certain universities deemed elite offer a curriculum divergent from that of their counterparts in low and middle-income countries (9). Students at these “elite” institutions gain exposure to new technologies and research incentives, which brings us to the realm of research. Remarkably, 75% of articles originating from developed nations account for just 25% of traumatic brain injury cases. This disparity highlights a significant research imbalance, and the common refrain underscores the need to bolster research capabilities in low-income countries (10). For neurosurgeons in the developing world, engaging in research often becomes a luxury due to multifaceted challenges. Financial barriers, including publication costs and paywalls for accessing articles, pose significant hurdles (11).

**Figure 1:**
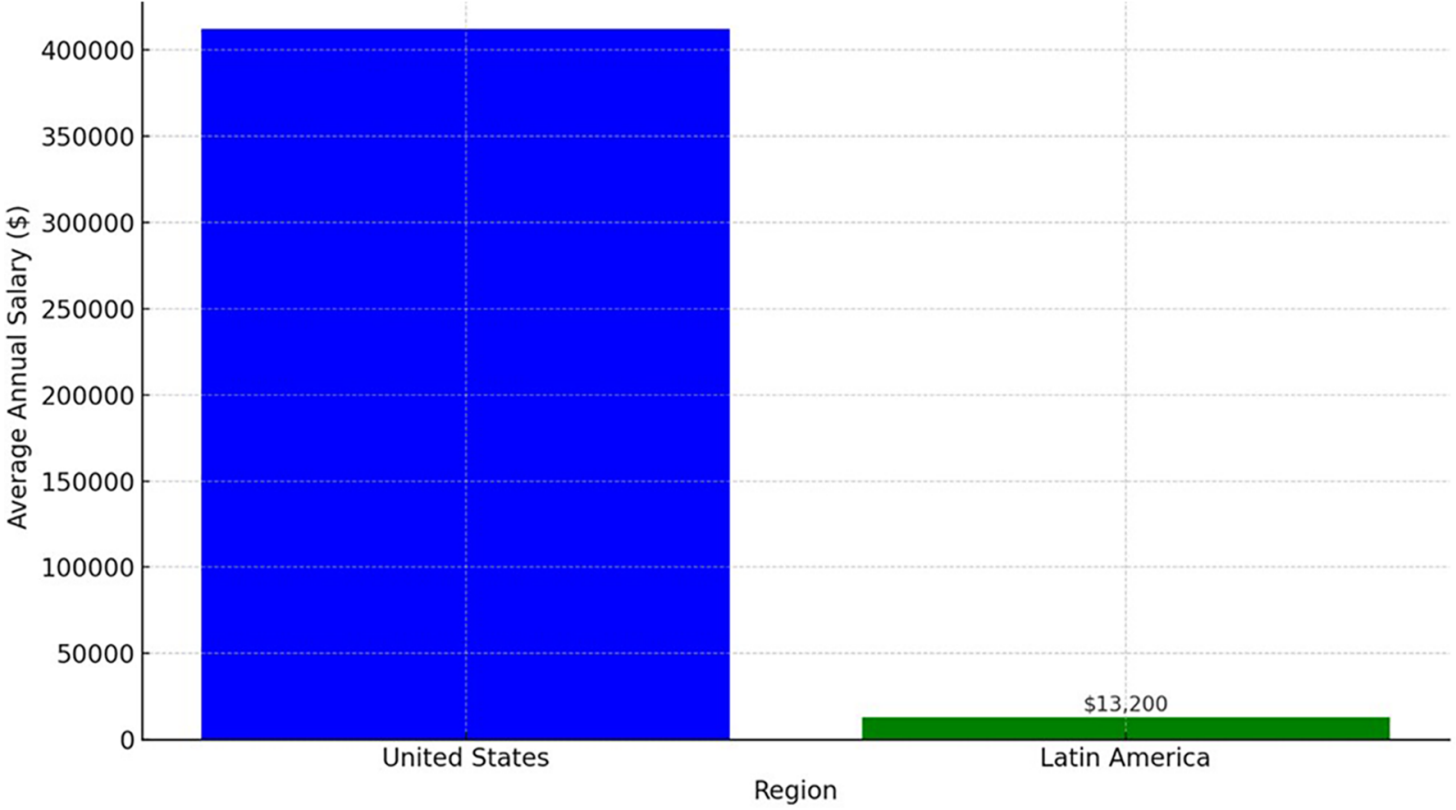
The vast differences in average annual salaries between neurosurgeons in the United States and Latin America region.

Comparing salaries between countries underscores the glaring divide according to “Neurosurgeon Salary in 2024|PayScale)” (12). Neurosurgeons in the United States receive a median? Salary of $412,000 dollars per year, compared to $13,200 dollars in Latin America, as of June 2023^1^ (Figure 1). Given such incongruities, the prospect of even attending conferences or workshops abroad remains difficult for neurosurgeons from developing nations. Research isn't cast aside due to a lack of interest but due to resource limitations. The present landscape demands reconsideration. High-impact journal publications often require substantial fees, and participation in conferences incurs significant costs. Could solutions emerge through free journal access for neurosurgeons in low-income countries? What if reduced fees facilitated their engagement in conferences and courses? In doing so, the realm of inclusion could be realized. Moreover, introspection is crucial. Are all articles published in high-impact journals genuinely contributing to advancing our practice? Is composing a manuscript easier in a high-income setting with financial backing and interdisciplinary team support? And what of the individuals responsible for paper approval? Addressing these questions demands honesty and transformation. Consequently, embracing change entails challenging conventions.

The shift toward inclusion could involve on-site verification of data sources instead of outright paper rejection. Substantive change may be catalyzed by rendering conferences and resources more accessible and supporting emerging nations' research aspirations. The call for global balance in neurosurgical progression resonates. Striving for equitable opportunities isn't merely a matter of fairness; it's a step toward elevating the entire field. By dismantling barriers and fostering inclusivity, we move closer to a neurosurgical landscape that thrives on the contributions of diverse minds, regardless of their geographical origins. In this pursuit, introspection is as vital as systemic transformation. Realizing a more inclusive and balanced neurosurgical landscape necessitates a multi-faceted approach. It involves shifting paradigms of evaluation, questioning long-standing biases, and reconsidering the infrastructure that perpetuates disparities. By challenging assumptions and embracing change, the field can progress toward a future where every neurosurgeon's potential can flourish, regardless of the socio-economic landscape they stem from, the journey toward equitable neurosurgical advancement involves peeling back layers of complexity. Beyond geographic origins, financial barriers, and established norms lies the potential for transformative change. It's a collective effort—an evolution—toward a field that truly reflects the diverse tapestry of talent and innovation present across the globe (13, 14).

### Heredity: a catalyst for change

Heredity plays a crucial role in shaping opportunities, extending beyond financial inheritance to include access to quality education, influential networks, and a privileged upbringing. This understanding isn't about undermining personal achievements but acknowledging the uneven playing field that not all aspiring neurosurgeons start from. Recognizing heredity's impact invites us to dismantle barriers and level the field, turning hereditary advantages into tools for advocating equality and uplifting those without the same head start (15). This approach fosters a culture of compassion, empathy, and action, encouraging mentorship and scholarship programs that offer support to those who might lack it. By addressing the complexities of heredity, we redefine success to focus on collective progress rather than individual accomplishments, ensuring every talent has the chance to thrive. Heredity, thus, becomes a catalyst for change, urging us to create a more inclusive and equitable future for all aspiring neurosurgeons, making the profession richer and more diverse. This narrative emphasizes collaboration and the power of leveraging one's position to benefit the broader community, shaping a neurosurgery field where everyone can succeed (16).

### Race and underrepresented minorities: a journey of equity

In the intricate mosaic of disparities, the brushstroke of race paints a compelling narrative that compels us to delve deeper into the very essence of our profession. Acknowledging the realities of racial inequalities is not an act of surrender, but rather a testament to our commitment to a more inclusive and just future within the realm of neurosurgery.

Race holds a potent influence, often steering the course of one's neurosurgical journey in ways that extend beyond individual merit. This acknowledgment is far from an invitation to victimhood; it's a call to acknowledge the historical biases and systemic hurdles that have, for too long, cast shadows on our field's potential for diversity and excellence (17).

The lens of race reveals disparities that intersect with every facet of neurosurgical pursuit. From medical school admissions to research opportunities, these disparities are a persistent undercurrent. The triumphs of some should not blind us to the realities faced by underrepresented minorities (URM), who often navigate a landscape marked by unspoken biases and barriers (18).

The journey toward equity is an inclusive one, embracing allies and advocates from all walks of life. When institutions, organizations, and individuals come together to address racial disparities, the impact ripples far beyond our immediate sphere. Collaborative efforts can empower the next generation of neurosurgeons, demonstrating that a diverse workforce not only benefits the field but also advances patient care (19).

**Figure 2:**
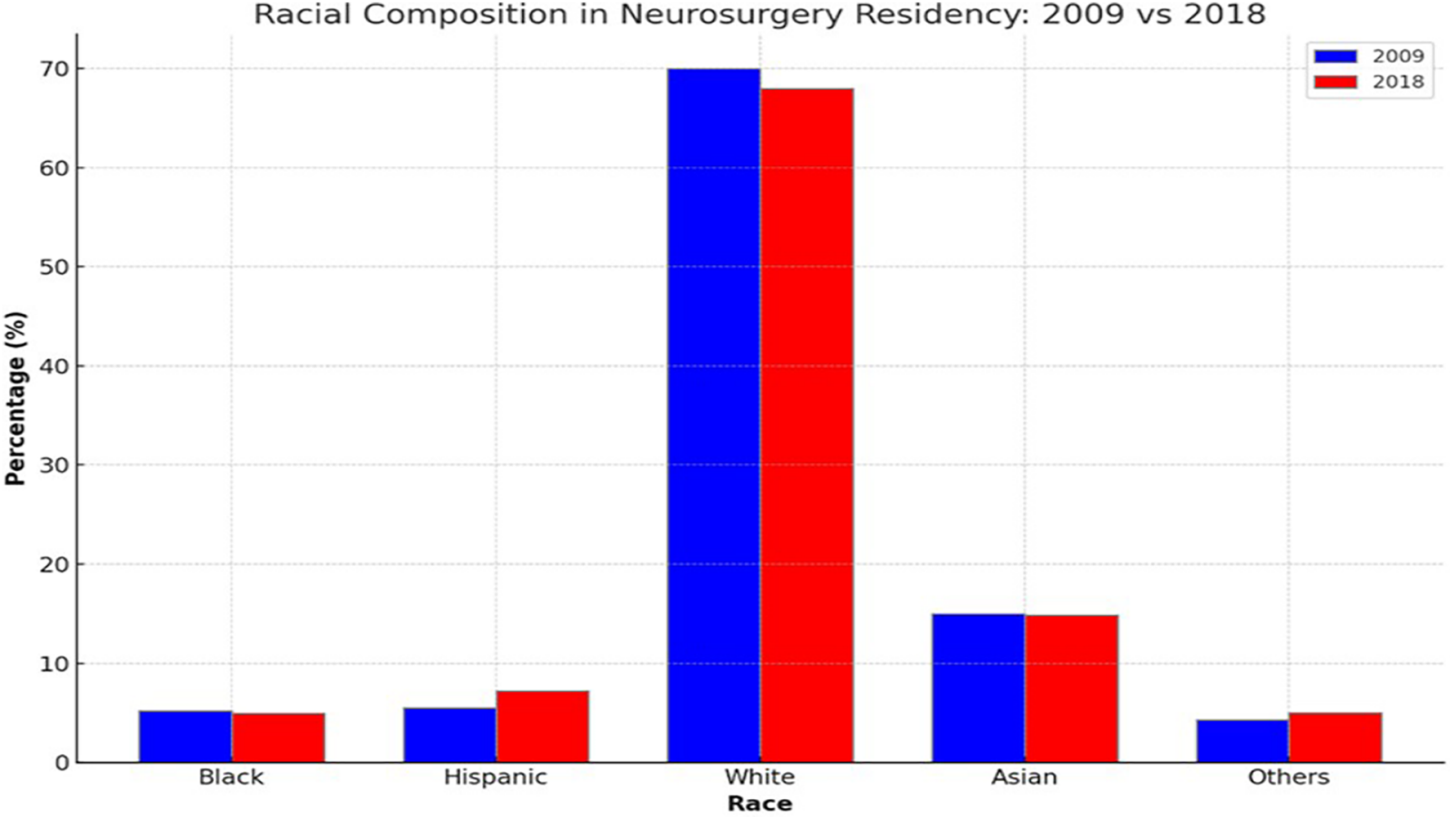
Comparison of racial composition of neurosurgeons in residence in US between 2009 and 2018.

Gabriel et al. in their study show that the percentage of Black and Hispanic applicants to neurosurgery program decreased across the observed period (4% and 1%, respectively). While Black people represented 5.2% of the resident pool in 2009, this decreased to 4.95% by 2018. Hispanic residents saw a <2% net increase (5.5%–7.2%) in resident representation but still fell behind when compared with census statistics. The application pool did not see a significant change in the percentage of White and Asian applicants; however, the percentage of residents did decrease slightly over the observed decade (20) (Figure 2).

Given the observed disparities in racial representation in the resident pool, a pertinent question is how meritocracy operates within this system. Are certain racial groups disproportionately affected by systemic barriers, preventing them from achieving the “merits” recognized by the selection process? Alternatively, are there biases present that affect the objective evaluation of candidates?

### Gender

“Neurosurgery is a specialty for men” is a regrettable expression heard several times. Diana Beck, from the United Kingdom (21) was the first female neurosurgeon in 1939 women are also the object of prejudices in the hiring, evaluation, promotion and compensation processes compared to men in neurosurgery, the high competitive ability, the long duration of the specialty, the arduous working hours in the operating room are qualities that in a context of meritocracy are traditionally considered positive values of the male stereotype, but not so of the female (22). “Stereotypes, very simple and internalized cognitive schemes that we apply to people because they belong to a group, such as gender, tell us how men and women should be. It is clear that there are men and women of many types, but stereotypes are placed at the extremes” (23), women in neurosurgery walk a tightrope: “They struggle not to seem too feminine, which could make them be considered” too weak according to what the stereotype says a woman should be, but not too dominant either, because this is accepted in the case of men, but generates antipathy in the case of women a fact that women do not have the same opportunities as men and that becoming a neurosurgeon is more difficult, a reflection of this disparity is the small number of women who hold the position of Professor in neurosurgery or head of a neurosurgery service compared to the large number of men who hold such positions, Stanford's Department of Neurosurgery faculty is nearly 25% female, an unprecedented level compared to other Neurosurgery programs around the United States, and the world (24). Feghali et al. conducted a study with a cohort of 1,255 male and 317 (20%) female trainees. Yearly trends indicated a significant drop in incoming female trainees in 2016, followed by significant increases in 2017 and 2019. On multivariable analysis, the following factors were associated with a higher average percentage of female graduates entering neurosurgery (25).

Diversity as an Asset, not a Quota: In a meritocratic system, diversity is recognized for its inherent value in enriching the field and improving patient care. However, it should not be reduced to a mere quota system. Instead, neurosurgical departments should focus on fostering an environment where diverse talents can flourish based on their merit.

### Socio-economic implications in the pursuit of meritocracy in neurosurgery

Educational Resources: Economic status can influence access to educational resources from early childhood through medical school. Students from higher economic backgrounds might have access to better schools, tutoring, test preparation resources, and extracurricular activities, all of which can contribute to a competitive application for neurosurgery residency. In the study by Kortz et al., it was observed that securing a training post in neurosurgery appears to be positively associated with graduates from top-tier medical institutions, predominantly those situated in the Northeast or Southern regions of the United States (26).

Medical School Prestige: Sometimes, the reputation of a medical school can influence residency selection. Students from economically disadvantaged backgrounds might have fewer options in choosing medical schools, which can inadvertently affect their chances during the matching process. Hovis et al. demonstrated that medical students who graduate from top 25 institutions, private medical schools, or those with an AANS chapter or an NSIG are more inclined to secure a position in esteemed training programs. This indicates potential inherent biases that program directors may need to address during the selection procedure (27).

Opportunities for Research and Networking: Pursuing research, attending conferences, and participating in internships or observerships can strengthen a candidate's application. However, these often require financial resources. Students with limited economic means may face challenges in accessing these opportunities, which can affect their competitiveness.

Hidden Costs: The training application process, including traveling for interviews, can be expensive. Candidates with limited financial resources might have to be selective in the programs they apply to or interview with, potentially limiting their opportunities; the total expenses for all components of the application process was US $10,255, in USA (28).

### Embracing the path forward: a call to unite for change

It's time to tackle the deep-rooted inequalities and make our community as diverse as the patients we serve. Acknowledging the biases and historical disparities is just the first step; real progress requires action. We need to create an inclusive culture where everyone's voice is heard and valued.

The push for change is urgent across all levels, from institutions to research labs. By supporting and mentoring those from underrepresented backgrounds, we can start to break down barriers. Working together, we can come up with better, more innovative solutions than we ever could alone. Diversity isn't just a nice idea—it's essential for innovation and for providing the best care to our patients (29).

Looking ahead, we remain inspired by the pioneers who saw beyond the limits of their time. Now, it's our turn to mentor the next generation, to reach across borders for new ideas, and to challenge old norms. By committing to this change, we can make neurosurgery a field where everyone, regardless of background, has the opportunity to succeed (19, 24).

### Strategies for prioritizing merit over other variables in neurosurgery

Promoting meritocracy in recruiting, hiring, and career advancement—especially in specialized fields like neurosurgery—requires a multi-pronged approach that addresses both systemic and individual-level factors, here are some strategies that could potentially help prioritize merit over other variables:

Transparent Recruitment Process
  Standardized Testing: Implementing uniform and transparent tests for evaluating both practical and theoretical skills can ensure that everyone is assessed using the same criteria.
  Anonymous Applications: Remove names, genders, and other identifying information from applications to minimize unconscious bias.
Equal Opportunities and Accessibility
  Financial Support: Offering scholarships or stipends can help candidates from lower economic statuses afford the costs associated with application and training.
  Internship Opportunities: For immigrants or underrepresented groups, targeted internship programs can offer valuable work experience and networking opportunities.
Fair Evaluation and Interviews
  Structured Interviews: Using a consistent set of questions for all candidates can help minimize the impacts of bias.
  Diverse Interview Panels: Including individuals from various backgrounds, departments, and ranks can help ensure a more balanced and fair evaluation process.
Training and Development
  Skill Development Programs: Continual professional development should be accessible to all, regardless of background.
  Mentorship Programs: Structured mentorship programs can help newcomers navigate the professional landscape and may help neutralize some of the disadvantages faced by underrepresented groups.
Monitoring and Feedback
  Data Analysis: Regularly collecting and analyzing data on the recruitment and advancement processes can help identify any patterns of discrimination or bias.
  Feedback Loops: Create anonymous channels for employees to provide feedback on the hiring and evaluation processes.
Policy and Guidelines
  Inclusive Language: Use inclusive language in job descriptions and during interviews to avoid discouraging any groups from applying or accepting positions.
  Legal Compliance: Ensure all policies are in line with existing laws and regulations concerning fair employment and equal opportunity.
Cultural Changes
  Bias Training: Regular training sessions can help staff recognize and counteract their biases.
  Open Dialogue: Encourage open discussions about diversity and inclusion, which can help create a culture that values merit over other variables.
  By implementing these strategies, institutions can make significant strides toward establishing a genuine meritocracy. It's important, however, to tailor these guidelines to the specific needs and challenges of the field. Specialized committees can be formed to oversee and adapt these policies as needed.

## Conclusion

Harmonizing Change: A Unified Vision for Tomorrow's Neurosurgery.

We underscore the journey towards a more inclusive and equitable future in neurosurgery as not just a goal, but a dynamic process fueled by resilience, collaboration, and a commitment to diversity. Disparities in the field, rather than being obstacles, are to be seen as opportunities for transformation and progress through understanding and strategic action. The narrative promotes a collective endeavor to dismantle barriers and embrace innovation, emphasizing the importance of mentorship, cross-institutional collaboration, and the amplification of underrepresented voices. This movement towards change is depicted as a symphony of efforts, where every participant plays a crucial role in building a neurosurgical community that values talent over background or identity. It calls for continued action in nurturing diversity, equity, and inclusivity, painting a future where every neurosurgeon has an equal and fair opportunity to contribute to the field. The aim is to inspire a shared commitment to forging a legacy of progress, mindful that we are the architects of a more harmonious and unified future in neurosurgery.

## Data Availability

The raw data supporting the conclusions of this article will be made available by the authors, without undue reservation.

## Footnotes

1 https://www.erieri.com/job/neurosurgeon.
